# Movement-based embodied contemplative practices: definitions and paradigms

**DOI:** 10.3389/fnhum.2014.00205

**Published:** 2014-04-14

**Authors:** Laura Schmalzl, Mardi A. Crane-Godreau, Peter Payne

**Affiliations:** ^1^Department of Family and Preventive Medicine, University of California San DiegoLa Jolla, CA, USA; ^2^VA San Diego Healthcare SystemLa Jolla, CA, USA; ^3^Department of Microbiology and Immunology, Geisel School of Medicine at DartmouthLebanon, NH, USA; ^4^Research and Development Service, Veteran's Administration Medical CenterWhite River Junction, VT, USA

**Keywords:** Yoga, Qigong, Somatics, embodiment, movement, contemplation, proprioception, mindfulness

## Abstract

Over the past decades, cognitive neuroscience has witnessed a shift from predominantly disembodied and computational views of the mind, to more embodied and situated views of the mind. These postulate that mental functions cannot be fully understood without reference to the physical body and the environment in which they are experienced. Within the field of contemplative science, the directing of attention to bodily sensations has so far mainly been studied in the context of seated meditation and mindfulness practices. However, the cultivation of interoceptive, proprioceptive and kinesthetic awareness is also said to lie at the core of many movement-based contemplative practices such as Yoga, Qigong, and Tai Chi. In addition, it likely plays a key role in the efficacy of modern somatic therapeutic techniques such as the Feldenkrais Method and the Alexander Technique. In the current paper we examine how these practices are grounded in the concepts of embodiment, movement and contemplation, as we look at them primarily through the lens of an enactive approach to cognition. Throughout, we point to a series of challenges that arise when Western scientists study practices that are based on a non-dualistic view of mind and body.

## Introduction

Compared to the extensive body of work on mindfulness-based practices, far fewer scientific studies have examined the mechanisms underlying movement-based embodied contemplative practices such as Yoga or Qigong. One likely reason is the inherent challenge of dealing with their multifaceted nature, typically involving specific movement sequences, specialized use of the breath, and modulation of attention (Wayne and Kaptchuk, 2008). Movement-based practices have, however, been shown to alleviate the symptoms of various clinical conditions (Jahnke et al., 2010; Wren et al., 2011), and elicit measurable changes in physiological stress markers (Lee et al., 2004; West et al., 2004), cognitive functioning (Manjunath and Telles, 2001; Silva et al., 2007), sensorimotor acuity (Kerr et al., 2008), as well as emotional states in healthy populations (Chattha et al., 2008). An important challenge for contemplative scientists is therefore to advance our understanding of the mechanisms underlying these complex practices. But what exactly constitutes a movement-based embodied contemplative practice (MECP)? Contemplative movement systems exist in almost all cultures of the world—from shamanistic dances, Christian liturgical gestures, and Eastern spiritual practices, to modern Western somatic practices. The initial challenge in looking at these systems is one of providing a taxonomy that is appropriate both to the systems themselves and to thorough scientific investigation. Whether a system is embodied or not, whether it is movement-based or not, and whether it is contemplative or not, are all relevant distinctions, so let us look at each of these aspects separately.

## Embodied

Over the past decades, cognitive neuroscience has witnessed a shift from predominantly abstract and computational views of the mind, to more embodied and situated views of the mind.

Francisco Varela (Varela et al., 1991) was one of the first to introduce the term “embodied mind” into cognitive neuroscience as a counter to the concept of a “disembodied mind,” a mental entity considered independently of its relationship to a body and the environment (for further perspectives see (Wilson, 2002), and (Ziemke, 2003). According to the “enactive approach” to cognition (Thompson and Varela, 2001; Thompson, 2005; Di Paolo and Thompson, 2014), living beings are autonomous agents that actively generate and maintain their physical and psychological identities, and that enact their cognitive domains through their activities. As such, the enactive approach postulates that human beings exists intrinsically as embodied beings, and that mental functions such as perception, cognition and motivation, cannot be fully understood without reference to the physical body as well as the environment in which they are experienced (Varela et al., 1991; Thompson, 2007).

The closely related “grounded theory” of cognition (Barsalou, 2008) posits that cognition (including abstract thought, conceptual knowledge and semantic memory) is grounded in the brain's modal sensory systems, rather than being merely based on abstract computations. Similarly, the dynamical systems approach to developmental theory (Camras and Witherington, 2005), psychoanalysis (Krueger, 2002) as well as the newly emerging body-oriented methods of psychotherapy (Heller, 2012), all emphasize that bodily sensory systems are the first to develop and that they play a fundamental role in the formation of the sense of self (Sheets-Johnstone, 1999). In sum, all these schools of thought converge on the fact that the experience of one's self in the world as a cognizant being does not solely emerge from neural activity within the brain. Instead, it involves a complex interplay of brain, body and environment, and the seamless integration of interoceptive, proprioceptive (including vestibular), kinesthetic, tactile, and spatial information (Ehrsson, 2007; Haselager et al., 2012; Ionta et al., 2011).

Within the field of contemplative science, the process of becoming reflectively attentive to bodily sensations and sensory experiences has so far been primarily studied in the context of seated meditation and mindfulness-based practices (Didonna, 2009). However, it is certainly at the core of many movement-based practices as well. In fact, systems such as Hatha Yoga, Qigong, Tai Chi, the Alexander Technique, and the Feldenkrais Method (see Supplementary Material for a brief description of these systems), all involve an explicit emphasis on attending to interoceptive, proprioceptive and kinesthetic qualities of experience. They also use concepts such as “being in one's body” to encourage an embodied experience of the self.

## Movement-based

One of the central motivations of examining movement within the context of contemplative practices can be related to the enactive approach described above. Movement is a fundamental characteristic of the embodied state, and the enactive as well as the grounded cognition approaches propose that the individual's capacity for self-movement and its underlying sensorimotor substrates are a constitutive part of all cognitive processes (Thompson and Varela, 2001; Barsalou, 2008). In addition, they propose that any embodied activity, including cognitive processes, takes the form of sensorimotor coupling with the environment. What a living organism senses and perceives is a function of how it moves, and how a living organism moves is a function of what it senses and perceives (Maturana and Varela, 1987). Along similar lines, Llinás (2002) echoes Sperry's earlier assertion (Sperry, 1952) that movement is the principal function of the nervous system, and that most advanced functions of the cortex can be seen as elaborations of the basic need to move toward or away from environmental stimuli. The word “emotion” is derived from the Old French “emouvoir” (“to stir up or agitate”), and from the Latin roots “ex-” (out) and “-movere” (to move). Similarly, the word “attention” is derived from the root “ten” (“to stretch out toward”) (Partridge, 1966). In that sense, feeling attracted or repulsed from something, or even directing or withdrawing ones attention from something, can be seen as subtle forms of movement (Day, 1964; Sheets-Johnstone, 1999).

For the most part, MECPs are based on internally generated self-willed movement (Krieghoff et al., 2011), and practitioners guide and adjust their movement based on subtle feedback from joints and muscles (Scott, 2012). Such voluntary and actively initiated movement, as opposed to externally evoked or purely passively imposed motion, is intrinsic to the sense of agency (Kalckert and Ehrsson, 2012), which in turn is central to the development of the sense of self (Thelen and Fogel, 1989). Given their emphasis on carefully executed intentional movements, we speculate that MECPs can be a tool for restructuring an individual's sense of agency, and consequently impact the exploration and transformation their sense of self. There are, however, also forms of MECPs that involve spontaneous movement that is not controlled voluntarily (e.g., Spontaneous Qigong, Tandava, Shaktipat, Katsugen-Kai, Latihan, and Kundalini (Louchakova and Warner, 2003). Such practices involve putting oneself into a state of receptivity and surrendering to the spontaneous movement that arises. Overtly, these movements may resemble animal movements, Taijiquan forms, or Yoga asanas and mudras. In other cases however, the “movement” is purely internal, involving vivid interior sensations of heat, vibration, “energy” currents, and even changes in experienced bodily shape and spatial extension. During infant development, spontaneous involuntary movement precedes controlled voluntary movement and the development of a sense of agency (Sheets-Johnstone, 1999). That is, an infant's experience of “movement occurs” precedes the experience of “I move” (Haselager et al., 2012). We suggest that MECPs involving spontaneous movement may therefore allow a constructive regression to early stages of development of voluntary motor control, and a consequent re-modeling of the sense of agency.

Asian movement-based practices such as Ashtanga Vinyasa Yoga (Jois, 1999) or Taijiquan (Jou and Shapiro, 1983) obviously involve overt voluntary physical motion, and strongly emphasize specific forms and qualities of movement. However, let us consider this quote from Yiquan master Wang Xiangzhai: “One should know that a big movement is not as good as a small movement, a small movement is not as good as stillness, one must know that only stillness is the endless movement.” (Wang, 2005). In Qigong practice, as in the internal martial arts, there is often a progression from an overt large motion, to a very small and subtle motion, to a purely internal or imagined movement—and this last is regarded as the most effective (as well as the most difficult) way of moving the “Qi” (life energy). So what needs to move, and to what extent does the movement need to be overtly evident, in order for a practice to qualify as movement-based? Here we propose the idea of extending the concept of “movement-based” so as to include very subtle, and even imagined, movement. This idea is supported by our knowledge of the neural mechanisms underlying motor control (Scott, 2012) and motor imagery (Schuster et al., 2011). Every execution of an overt movement controlled by the primary motor cortex is preceded by activations in premotor and supplementary motor areas. This preparatory phase has the purpose of organizing the movement by setting the appropriate posture, muscle tone and autonomic tone, and allow for integration of information from other cortical regions (e.g., visual, somatosensory, executive, affective and motivational) into the intended motor action (Wolpert et al., 2011). Moreover, premotor, supplementary motor as well as cerebellar areas are strongly activated during motor imagery without any actual physical movement (Gerardin et al., 2000), which in itself can improve motor skills and even physical strength (Yue and Cole, 1992; Sharma et al., 2006; Schuster et al., 2011). Qigong uses a technique very similar to motor imagery referred to as “moving the Qi.” It consists of moving one's attention through the body so as to create a sensation of a flowing current of energy (Johnson et al., 2000). This form of practice is regarded by most practitioners as essential to the benefits of Qigong. We postulate that such subtle movement may have the effect of re-programming counterproductive patterns of intention and action by bringing the process of initiating action up to full consciousness.

At this point it might appear that every contemplative practice involves movement to some extent, and in fact we would argue that this is the case. Even in the most static forms of seated meditation the whole body is in constant subtle motion with the rhythm of the breath. That said however, we propose to define a movement-based practice as one in which the principal focus is on the intentional induction, or the intentional disinhibition, of overt movement or subtle internal sensations of movement. This definition excludes simply watching the breath while seated, unless specific emphasis is put on cultivating attention to the subtle movement that accompanies the breath.

## Contemplative

In recent scientific explorations of contemplative practices, the terms “contemplation” and “meditation” have often been used interchangeably. The dictionary definition of contemplation includes both secular and religious meanings, referring to sustained attention and deep consideration of an object of interest that is often used in the context of religious or spiritual experience. The root of the word is the same as that of “temple”—“tem” or “to cut”—implying the concept of “carving out” a special time and place apart from daily preoccupations (Partridge, 1966). The word meditation has its origins in the Indo-European root “med,” implying the concepts “to measure,” “to consider,” or “to think about.” In Western Christian tradition “contemplation” refers to non-conceptual awareness of the Divine, whereas “meditation” carries the implication of conceptual, thought-based consideration of religious ideas. In the encounter between Western thought and Asian religions however, the English word meditation has come to be used to denote a wide range of diverse practices (for a discussion of this issue in relation to early Buddhist writings see Brooks, 2004).

A theoretical approach that is fundamental to the investigation of contemplative and meditative practices is neurophenomenology (Varela, 1996; Lutz and Thompson, 2003), an offshoot of the enactive approach described above. Neurophenomenology emerged out of the need to make systematic use of first-person methods and introspective phenomenological reports in the study of subjective conscious experience, and to relate the information gathered through those methods to complex dynamical systems analysis of brain activity. First-person methods, whether phenomenological, meditative or psychotherapeutic, are specifically aimed at increasing an individual's sensitivity to the quality of their experience from moment to moment. As a result, they have the potential to enable tacit, pre-verbal and pre-reflective aspects of the experience that typically remain merely “lived through,” to become subjectively accessible. First-person methods exist within several contemplative practices and traditions that systematically cultivate the capacity for attentive self-awareness. While the individual practices differ in terms of specifically adopted techniques, they typically involve a disciplined process of becoming reflectively attentive to experience. In phenomenology this process is known as “epoché” (Husserl, 2012), and is described as three intertwining phases that form a dynamic cycle. In a nutshell, the three phases consist of suspension of habitual thoughts, redirection of attention to the experience itself, and receptivity to whatever arises from it (Depraz et al., 2000). In the context of MECPs, the redirection of attention predominantly entails cultivating awareness of bodily sensations and proprioceptive feedback related to the specifically employed movement and breathing techniques.

In recent years there has been an increasing interest in trying to understand the neural mechanisms underlying contemplative states. In fact, mindfulness-based meditation practices are now known to engage selective brain areas and neural networks involved in attention, body awareness, emotion regulation and the sense of self (Hölzel et al., 2011; Kerr et al., 2013). In addition, meditative practices have also been found to be typically associated with altered activation of the so-called default mode network (DMN) (Raichle et al., 2001), and enhanced connectivity between cortical regions implicated in self-monitoring and cognitive control (Brewer et al., 2011). The DMN is known to be involved in the construction of the “autobiographical self,” and the assessment of stimuli for their relevance to the mentally sustained image of oneself. The altered activity of the DMN during meditative states has therefore been proposed to underlie a shift to less self-centered and more objective awareness of interoceptive as well as exteroceptive sensory events (Brewer et al., 2011). To which extent these finding also relate to MECPs remains an open question. Yoga and Qigong are broad and multifaceted categories, both including a range of “meditative movement” practices (Larkey et al., 2009), which are typically complemented by seated meditation techniques. Hence, while many of the already formulated theories about the mechanisms underlying traditional mindfulness-based practices might also apply to MECPs, it can also be assumed that MECPs may involve additional distinct mechanisms. Specifically, since movement increases the intensity of proprioceptive stimuli (Prochazka, 2011), it is possible that MECPs may offer a more efficient form of practice than seated meditation when it comes to cultivating bodily awareness and the sense of self.

Regardless of the specific underlying mechanisms, for most practitioners of MECPs contemplation represents the cultivation of sustained attention and equanimity (Desbordes et al., 2014), that for some has the ultimate aim of transforming habitual self-identity (akin to most seated meditation practices). However, MECPs can also be practiced without specific reference to altered self-identity as such. For example, Hatha Yoga can be practiced as a form of physical therapy, or Taijiquan purely as a martial art. Whether in such cases these types of practices can still be defined as contemplative remains subject to interpretation. In any case, the wide spectrum of potential applications of MECPs underlines the importance of documenting precisely which of their component parts are emphasized within a specific intervention—not only for the sake of scientific clarity, but out of respect to the systems themselves.

## Additional considerations

So far we have referred to MECPs as performed by an individual person. However, there are also forms of MECPs involving two people. The relationship between these two may be that of master and disciple, teacher and student, therapist and client, or co-practitioners. Together, they enter a state of enhanced connectivity referred to as “resonance” (Siegel, 2007; Nummenmaa et al., 2012). In this state there is a largely automatically occurring sharing of affective and somatosensory experience, said to also involve a simultaneous activation of affective and sensory brain structures in both individuals. These phenomena have been documented in recent research in social neuroscience (Singer and Lamm, 2009). This form of dyadic contemplation is at the core of many Eastern movement-based systems (Wallace, 2001), as well as Western systems such as the Feldenkrais Method, the Alexander Technique, and body-oriented psychotherapy.

A further example of a novel therapeutic system that might fall into the category of MECPs is Somatic Experiencing (SE). Based on observations of how animals in the wild recover from trauma, its principal method is based on attention to bodily (interoceptive, proprioceptive, kinesthetic, tactile and spatial) sensations (Levine, 1997). According to SE theory, it is proposed that the practitioner is guided by the resonant relationship and verbally leads the client into internal and external movement, enabling a rebalancing of the autonomic nervous system (Levine, 2010).

## Conclusion

As pointed out in a seminal article by Kerr (2002) and more recently by Payne and Crane-Godreau (2013), several challenges arise when Western scientists study practices that stem from ancient Eastern traditions. These authors underline the importance of having a thorough understanding of a system in its own terms before attempting to interpret it from a modern scientific perspective. They point out the risk of attempting to shoehorn a system into an already existing conceptual framework, which often eliminates the possibility of genuinely new discovery.

So what does it mean to look for the mechanisms underlying MECPs? Most of these practices have their own intrinsic complex bodies of theory. As both scientists and practitioners of MECPs, we are naturally drawn to wanting to operationalize them and understand them in our own “language.” In doing so however, our aim is not to replace their already existing frameworks with new scientific explanations (Smolin, 2013). Rather, our task is one of translation—the translation of phenomena and theories emerging from one world-view into a language based on a very different world-view. Neither of these languages is right or wrong, but each of them has advantages and disadvantages. If neuroscience will remain open to encountering phenomena not previously recognized, this will undoubtedly improve our scientific understanding of human functioning and of how ancient practices can enhance human wellbeing in our modern times.

### Conflict of interest statement

The authors declare that the research was conducted in the absence of any commercial or financial relationships that could be construed as a potential conflict of interest.
