# Supplementary material for: Movement-based embodied contemplative practices: definitions and paradigms
Source: Front Hum Neurosci. 2014 Apr 14;8:205. doi: 10.3389/fnhum.2014.00205 (PMC3995074; doi:10.3389/fnhum.2014.00205)
Supplement: Supplementary file 1 [file DataSheet1.PDF]

Movement-based embodied contemplative practices: Definitions and paradigms

Laura Schmalzl[1,2]*, Mardi A. Crane-Godreau[3,4]* & Peter Payne[3]*

[1]Department of Family and Preventive Medicine, University of California San Diego, CA, USA
[2]VA San Diego Healthcare System, CA, USA
[3]Microbiology and Immunology, Geisel School of Medicine at Dartmouth, Lebanon, NH, USA
[4]Research and Development Service, Veteran's Administration Medical Center, White River Junction, VT, USA

*These authors contributed equally to this work

Correspondence:

Laura Schmalzl, PhD

Department of Family and Preventive Medicine
School of Medicine
University of California San Diego
9500 Gilman Drive
La Jolla
CA 92093
USA

E-mail: lschmalzl@ucsd.edu

Supplementary Material

Below is a brief description of a selected list of movement-based embodied contemplative practices (MECPs). We would like to note that the list is by no means exhaustive and that there are numerous additional practices that can be classified as MECPs. Similarly, the descriptions are purposely succinct and meant to serve just as a general overview.

Yoga

The discipline of Yoga refers to a series of physical, mental, and spiritual practices originating in ancient India as one of six orthodox schools of dharmic Hindu philosophy. The term Yoga stems from the Sanskrit root “yui” (“to yoke” or “to concentrate”), and may refer to the union of the individual with the divine, the union of body, mind and spirit, or more simply the yoking of attention which ordinarily fluctuates from one object to the other. The most well-known foundational text of Yoga is Patanjali’s Yoga Sutras (Satchidananda, 1978). Patanjali defines the primary aim of Yoga as achieving a state of “cessation of the fluctuations of the mind”, with the ultimate goal of transcending discursive knowledge and habitual self-identity, and the consequent obtainment of permanent peace. The past century has witnessed the emergence of numerous Yoga “styles” in both Eastern and Western cultures, an evolution that makes it often challenging to distinguish genuinely traditional practices from modernized versions (Singleton, 2010). Most modern Yoga inspired practices have a strong emphasis on physical asanas or postures, and are often referred to under the umbrella term of Hatha Yoga (Akers, 2002). Subtypes of Hatha Yoga include Viniyoga, Ashtanga Vinyasa Yoga, Iyengar Yoga, Kundalini Yoga, and Yin Yoga. While these systems differ in their individual characteristics, they all involve specific postures or movement sequences, specialized use of the breath and cultivation of focused attention.

Qigong

The word “Qigong” (“work on the breath” or “life energy”) is of relatively recent origin, having come into usage in the mid-20th century among an early Communist cadre. Prior to that however, various techniques of Qigong existed in a wide range of religious, medical and martial contexts with a variety of designations. It is therefore important to differentiate between these traditional systems themselves, and the complex social and cultural purposes they have been used to promote (for an in-depth study of these issues see Palmer, 2007). As presently used in the West, the term Qigong refers to a very broad range of traditional Chinese practices. There are four principal forms of Qigong, which are primarily aimed at health maintenance, medical treatment, martial skill, and spiritual development respectively. The common basis shared by all forms of Qigong is the use of static postures, simple movements and breathing techniques, accompanied by attention to bodily sensations and specific visualizations. The main aim of Qigong practices is the restoring of a free flow of “Qi” (“life energy”) (Cohen, 1999).

Taijiquan

Taijiquan, also spelled T’ai Chi Ch’uan and popularly abbreviated as Tai Chi, is a martial art based on Chinese philosophical principles of Yin and Yang. It is sometimes classified as a martial form of Qigong, although its exact origin is controversial (Wile, 1996). Taijiquan has become very popular world-wide, and today it is mostly practiced as a health-focused and contemplative discipline with little reference to its martial applications (Jou & Shapiro, 1983).

Feldenkrais Method

The Feldenkrais Method (Feldenkrais, 1990; Rywerant & Feldenkrais, 2003) includes two systems: Awareness Through Movement, which consists of verbally guided patterns of complex voluntary movement, and Functional Integration, which consist of hands-on treatment involving gentle touch, guided movement, and verbal instruction. The main aim of the Feldenkrais Method is to improve patterns of motor action by providing the motor system with an experience of a wide range of alternate paths for action. Improved motor patterns are in turn assumed to have a positive impact on many other aspects of a person's functioning.

Alexander Technique

Developed by Frederick Matthias Alexander (Alexander & Maisel, 1989; Barlow 1973) towards the end of the 19th and beginning of the 20th century, the Alexander Technique is a method of mind-body re-education. It emphasizes the voluntary inhibition of dysfunctional automatic postural responses, and the concomitant conscious substitution of an improved functional pattern. Although overtly focused on physical posture and movement, it is aimed at affecting all aspects of the self, including cognitive functioning and emotional states.
